# Triggering through NOD-2 Differentiates Bone Marrow Precursors to Dendritic Cells with Potent Bactericidal activity

**DOI:** 10.1038/srep27263

**Published:** 2016-06-06

**Authors:** Nargis Khan, Mohammad Aqdas, Aurobind Vidyarthi, Shikha Negi, Susanta Pahari, Tapan Agnihotri, Javed N. Agrewala

**Affiliations:** 1 CSIR-Institute of Microbial Technology, Chandigarh, 160036, INDIA

## Abstract

Dendritic cells (DCs) play a crucial role in bridging innate and adaptive immunity by activating naïve T cells. The role of pattern recognition receptors like Toll-Like Receptors and Nod-Like Receptors expressed on DCs is well-defined in the recognition of the pathogens. However, nothing is precisely studied regarding the impact of NOD-2 signaling during the differentiation of DCs. Consequently, we explored the role of NOD-2 signaling in the differentiation of DCs and therefore their capability to activate innate and adaptive immunity. Intriguingly, we observed that NOD-2 stimulated DCs (nDCs) acquired highly activated and matured phenotype and exhibited substantially greater bactericidal activity by robust production of nitric oxide. The mechanism involved in improving the functionality of nDCs was dependent on IFN-αβ signaling, leading to the activation of STAT pathways. Furthermore, we also observed that STAT-1 and STAT-4 dependent maturation and activation of DCs was under the feedback mechanism of SOCS-1 and SOCS-3 proteins. nDCs acquired enhanced potential to activate chiefly Th1 and Th17 immunity. Taken together, these results suggest that nDCs can be exploited as an immunotherapeutic agent in bolstering host immunity and imparting protection against the pathogens.

Dendritic cells (DCs) are sentinels of adaptive immunity and exhibit tremendous versatility in their function. They present antigens to naïve T cells and deliver optimum signals for their activation^1^. Despite of their crucial role in the generation of adaptive immunity, *Mycobacterium tuberculosis (Mtb)* infected DCs are less efficient in constraining the growth of the bacterium than macrophages and hence they are considered inferior in bactericidal activity^2^. However, recently a distinct subset of DCs with specialized innate machinery such as production of TNF-α and iNOs; TNF-α/inducible nitric oxide synthase (iNOs)-producing DCs (tip DCs) has been identified from the spleen of *Listeria monocytogens* infected mice^3^. These DCs are endowed with a property to clear infection. Another novel subset of DCs expressing 6-sulfo LacNAc and exhibiting proinflammatory function has been documented in human peripheral blood, producing large amount of TNF-α in response to lipopolysaccharide (LPS)^4^. Despite of the fact that these DCs have potent innate property and play a crucial role in restricting the growth of pathogens, not much has been elucidated about the factors that govern their differentiation and activation. Emerging evidences suggest that environmental milieu or inflammation specific to pathogens decide the fate of DCs precursor to acquire distinct functional subtypes like stimulatory DCs and regulatory DCs^5^. The stimulatory DCs are activated by pathogens and induce effective immune response by activating adaptive immunity and skewing T cell response towards Th1, Th2, or Th17 phenotypes. However, regulatory DCs are induced by tolerogenic environment during autoimmune diseases. These DCs suppress T cell activation and proliferation and provide signals that enable Tregs differentiation and expansion^5^. For example TGF-β promotes the development of Langerhans like cells^6^,^7^. Langerhans like cells can process and present antigen and maintain immune homeostasis in the skin by activating resident regulatory T cells^8^. Exposure to IL-6, IFN-γ and IL-32 modulate the pathways involved in the differentiation of DCs^9^,^10^. Mycobacterial latency associated alpha-crystalline protein (Acr-1) impairs DCs maturation and function^11^.

In addition to cytokines, innate ligands have been implicated to modulate the differentiation of DCs. TLR-2 triggered monocytes derived Langerhans like cells (mLCs) secrete IL-1β, TGF-β and IL-23 and helps in the differentiation of Th17 cells against bacterial insult^12^. TLR-7/TLR-8 agonists impair the differentiation and maturation of DCs^13^. Further, LPS blocks the differentiation of monocytes to DCs *in vivo*^14^. In addition, other pattern recognition receptors (PRRs) like Nucleotide-binding oligomerization domain like receptors or NOD-like receptors (NLRs) are known to play strategic role in regulating DCs activity. Recently, NOD-2 which is an important member of NLRs family has been acknowledged in regulating the DCs activity with better efficiency to present antigen to CD8 T cells^15^. However, nothing has been documented about the role of NOD-2 signaling in the differentiation of DCs. NOD-2 consists of an N-terminal caspase recruitment domain (CARD), an intermediate NOD and a C-terminal leucine-rich repeats (LRRs) domain. It recognizes the peptidoglycan derivatives muramyl dipeptide (MDP). N-glycolyl muramyl dipeptide (NGMDP) is a modified agonist of NOD-2 with significantly better stimulatory activity than MDP^16^,^17^. It is important to mention that NOD-2 is expressed in the cytoplasm and to ligate with receptors, a ligand for NOD-2 is internalized into acidified vesicles by endocytosis dependent on the clathrin and dyamin pathways^18^,^19^. Here, we explore the influence of signaling of NOD-2 with NGMDP in the activation and differentiation of DCs with improved functions. We observed that nDCs exhibit activated phenotype with high expression of costimulatory molecules, abundant release of cytokines and NO. Importantly, nDCs were highly efficient in restricting the intracellular growth of *Mtb*. This study for the first time unveils the role of NOD-2 in the differentiation of the DCs, which make their contribution more specialized towards innate immunity.

## Results

### Signaling through NOD-2 influences the differentiation of DCs

**Figure 1:**
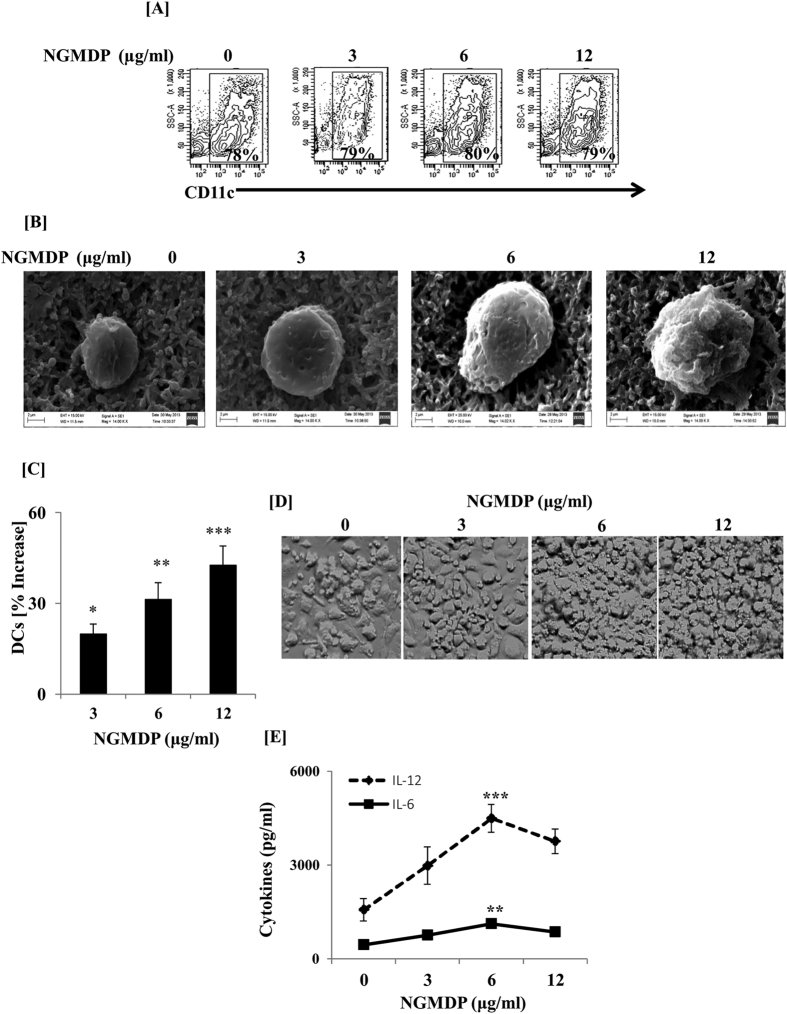
Signaling through NOD-2 bolstered the differentiation of DCs. BMCs were cultured in the presence of different doses of NGMDP, a ligand for NOD-2 along with GMCSF + IL-4 for 6 d. Later, cells were monitored for the (**A**) expression of CD11c by flowcytometry and (**B**) size of nDCs was determined by SEM (magnification: 1400 KX); (**C**) percentage increase in the yield of DCs was calculated with respect to control DCs, as determined by trypan blue exclusion method; (**D**) enhancement in the number of nDCs was evidenced by bright field microscopy (magnification: 40X). Images displayed are from 5–6 fields; (**E**) IL-6 and IL-12 were estimated in the SNs by ELISA. Control cultures comprises of DCs cultured in the absence of NGMDP. Data shown are representative of 3 independent experiments. The results are expressed as mean ± SD. nDCs indicate BMCs differentiated into DCs in the presence of NGMDP. *p < 0.05, **p < 0.01, ***p < 0.001.

Bone marrow cells (BMCs) were cultured in the presence of GMCSF + IL-4 along with NGMDP, a ligand for NOD-2 (NOD-2L). Triggering through innate ligands blocks the differentiation of BMCs to DCs. Therefore, we first studied the expression of CD11c, which is a classical marker of DCs. We observed that DCs cultured with NOD-2L (nDCs) showed a similar expression of CD11c, as control DCs (DCs differentiated in the presence of GMCSF + IL-4) (Fig. 1A). However, these cells showed enlargement in the size, as demonstrated by scanning electron microscopy (Fig. 1B). We also noted a significant (p < 0.01) expansion in the yield of nDCs (Fig. 1C). This information was corroborated by bright field microscopy (Fig. 1D). Furthermore, nDCs exhibited substantial enhancement in the release of IL-6 (p < 0.01) and IL-12 (p < 0.001) than control DCs (cDCs) (Fig. 1E). All the observed changes in the nDCs were noticed in a dose dependent fashion (Fig. 1A–E). Since optimum changes in the functionality (release of IL-6 and IL-12) of nDCs were observed at 6 μg/ml of NGMDP, hence this dose was used in all subsequent experiments.

### DCs acquired mature phenotype on signaling through NOD-2

**Figure 2:**
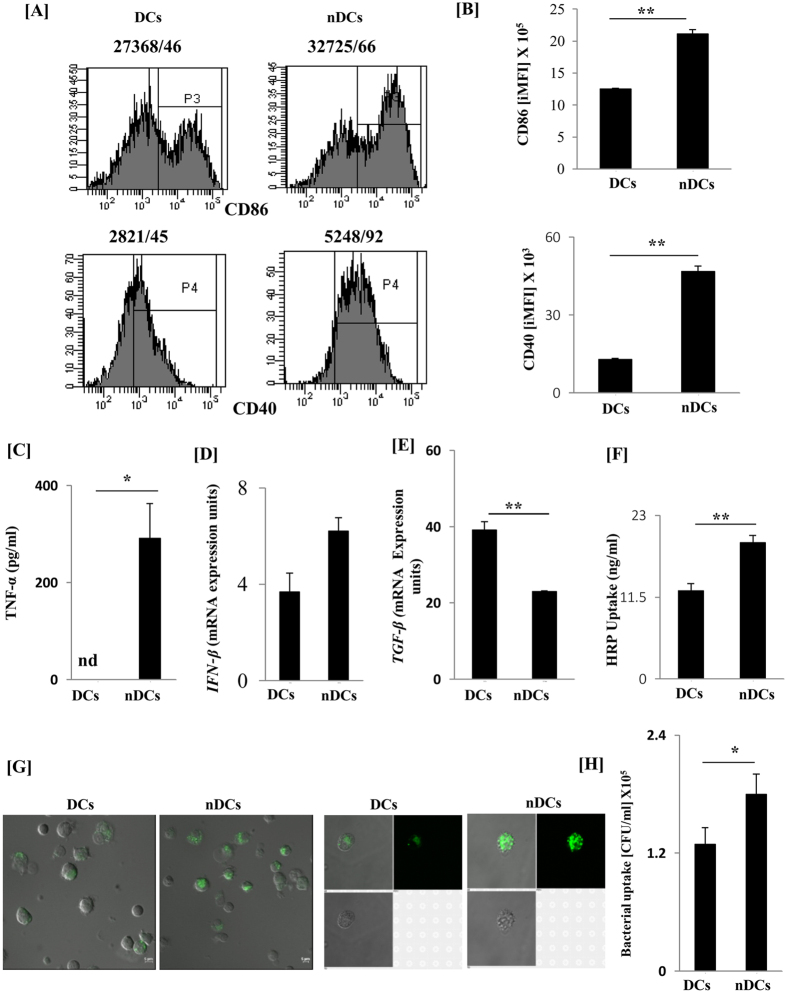
DCs activated through NOD-2 displayed greater potential for phagocytosis. (**A**) SSC^+^ CD11c^+^ nDCs were stained for the expression of CD86 and CD40 by flow cytometry. For analysis of CD40 and CD86, cells were gated on SSC^+^ and CD11c^+^cells after the FSC and SSC gate was set. The numbers in the inset indicate MFI/% [make to iMFI] population; (**B**) the bar diagrams depict the iMFI; (**C**) culture SNs were assessed for TNF-α by ELISA; (**D**,**E**) mRNA expression of IFN-β and TGF-β was quantified by RT-qPCR; (**F**) HRP and (**G**) dextran-FITC uptake by nDCs was demonstrated by colorimetry and confocal microscopy, respectively. Data were normalized with control cells kept on ice. (**H**) nDCs were infected with *Mtb* for 4 h. Later, phagocytosis of bacterium was assessed by CFU/ml. Results are expressed as mean ± SD. Data shown are representative of 3 independent experiments. *p < 0.05, **p < 0.01.

Efficiency of DCs depends preferably on three important factors: i) the expression of MHC and costimulatory molecules and release of cytokines; (ii) migration from periphery to the T cell areas of lymphoid organs to prime T cells; (iii) antigen uptake, processing and presentation to activate and differentiate naïve T cells. Interestingly, we observed that nDCs showed highly activated phenotype, as supported by a significant augmentation in the expression of CD86 (p < 0.01) and CD40 (p < 0.01) than cDCs (Fig. 2A,B). Further, abundant production of TNF-α (p < 0.05) by ELISA and considerable change in IFN-β was noted by RT-qPCR (Fig. 2C,D). In contrast, we observed significant decline in the levels of immunosuppressive cytokine TGF-β (p < 0.01) at mRNA level by RT-qPCR (Fig. 2E). Excitingly, nDCs exhibited significant (p < 0.01) enhancement in pinocytosis of the soluble antigen (HRP) (Fig. 2F) and phagocytosis of dextran-FITC and *Mtb* (p < 0.05) by confocal microscopy and CFUs, respectively (Fig. 2G,H). These results indicate that despite of highly activated phenotype, nDCs are efficient in antigen uptake.

### nDCs efficiently respond to innate stimuli

**Figure 3:**
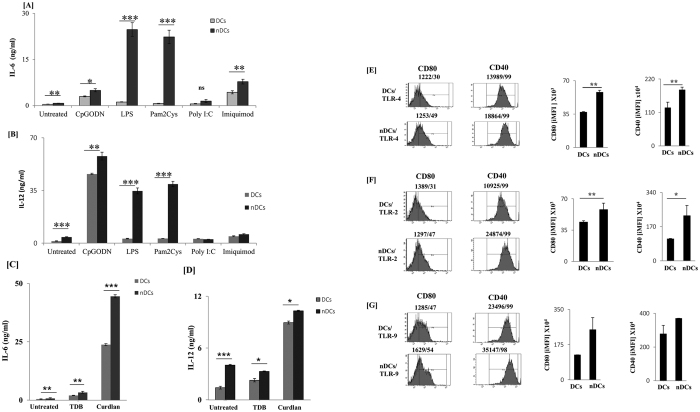
nDCs are not tolerized DCs since they responded efficiently to innate stimuli. nDCs were stimulated with LPS EK ultrapure: 5 ng/ml (TLR-4L), Pam2Cys: 2.5 ng/ml (TLR-2L), CPG-ODN: 2 μg/ml (TLR-9L ), imiquimod: 1 μg/ml (TLR-7L ), poly I:C: 10 μg/ml (TLR-3L ), curdlan 10 μg/ml (dectin-1L), TDB: 2 μg/ml (mincle L ) for 24 h. Later, (**A**–**D**) SNs were harvested and IL-6 and IL-12 were estimated. nDCs were stimulated with (**E**) TLR-4; (**F**) TLR-2; (**G**) TLR-9. The cells were assessed for the expression of CD80 and CD40 by flow cytometry. Number in the inset indicates the MFI/% of nDCs (left panel). Bar graph depicts integrated MFI (iMFI) (right panel). Data shown as mean ± SD are representative of 3 independent experiments. *p < 0.05, **p<0.01, ***p < 0.001.

Next, we were curious to know if nDCs have not undergone tolerization and can still respond to other innate stimuli like TLRs. Noteworthy, nDCs showed significant improvement in the production of IL-6 and IL-12 than cDCs on signaling through different TLRs using their respective ligands; LPS for TLR-4 (IL-6: p < 0.001, IL-12: p < 0.001), Pam2Cys for TLR-2 (IL-6: p < 0.001, IL-12: p < 0.001), CpGODN for TLR-9 (IL-6: p < 0.01, IL-12: p < 0.05) and imiquimod for TLR-7 (IL-6: p < 0.05) (Fig. 3A,B). No change was observed in the case of poly I:C, a ligand for TLR-3. Further, we examined the effect of curdlan and trehalose-6, 6-dibehenate (TDB), a ligand for dectin-1 and mincle (C-type lectin receptors), respectively. Results showed the abundant release of IL-6 (p<0.001) and IL-12 (p < 0.05) by nDCs than cDCs (Fig. 3C,D). Furthermore, triggering of nDCs through TLR-2 and TLR-4 showed upregulation of CD80 (p < 0.001) and CD40 (p < 0.001), as compared to control DCs (Fig. 3E,F). However, a marginal increase was noted with TLR-9 stimulation (Fig. 3G). These results signify the non-tolerogenic nature of nDCs.

### Signaling of DCs through NOD-2 enhanced the release of NO

**Figure 4:**
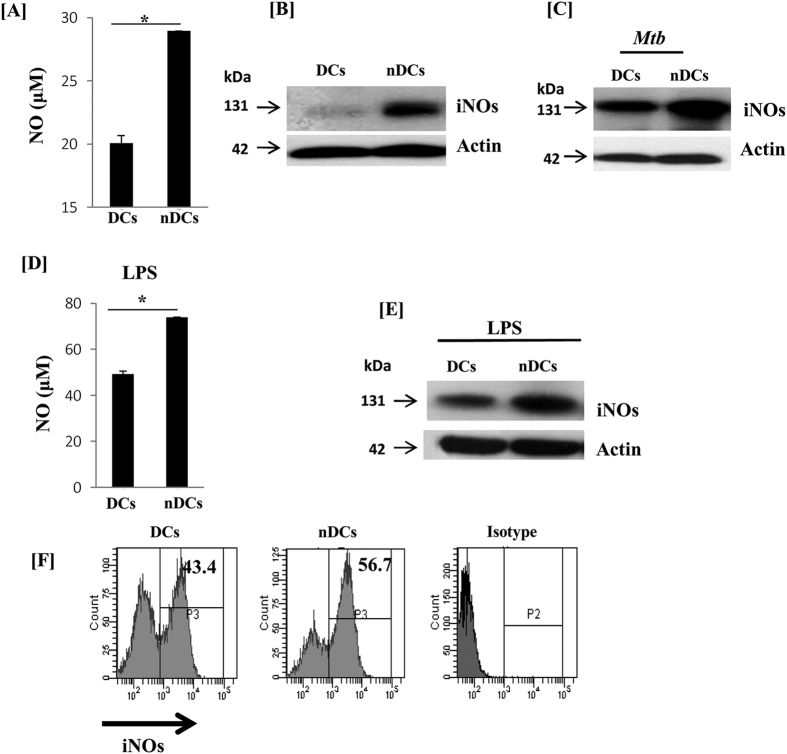
nDCs produced significantly higher NO in response to *Mtb* infection. (**A**) NO was estimated in the SNs of nDCs by Griess assay; (**B**) iNOs was detected in whole cells lysate of nDCs. (**C**) nDCs infected with *Mtb* were cultured for 24 h and iNOs was detected by Western blotting; actin was used as a loading control. nDCs were treated with LPS for 24 h (**D**) NO was estimated in the SNs; iNOs was detected by (**E**) Western blotting and (**F**) flow cytometry. For analysis of iNOS, cells were gated on SSC^+^ and CD11c^+^cells after the FSC and SSC gate was set. Data shown as mean ± SD are representative of 3 independent experiments.*p < 0.05.

nDCs showed highly matured and activated phenotype. Therefore, we next investigated whether nDCs exhibit improved bactericidal activity than cDCs. Firstly, we measured NO since it is considered as a potent molecule responsible for bactericidal activity^20^. We noticed increase in the (p < 0.01) production of NO (Fig. 4A). Further, this was corroborated by detecting increased expression of iNOs (Fig. 4B). Similarly, higher expression of iNOs was also detected in the *Mtb* infected nDCs (Fig. 4C). Furthermore, these results were validated by greater production of NO (Fig. 4D) and expression of iNOs by LPS stimulated nDCs through colorimetric method, Western blotting and flow cytometry (Fig. 4D–F).

### nDCs inhibit *in vivo* growth of intracellular pathogens

**Figure 5:**
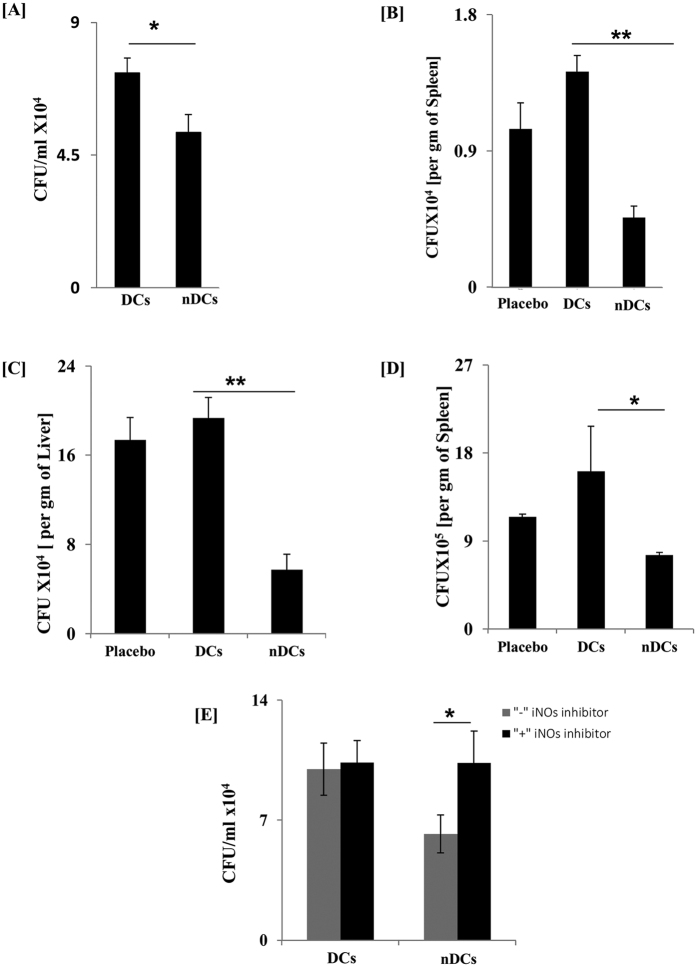
nDCs restricted in vitro and in vivo survival of intracellular *Mtb*. (**A**) nDCs were infected with *Mtb* (MOI 1:5) for 4 h. Survival of *Mtb* was determined after 24 h by CFUs. Bar graph depicts the CFU/ml of mean ± SD of the quadruplicate wells. (**B**) nDCs were adoptively transferred into mice. After 5 d, animals were challenged with (**B**) *Mtb* (**C**,**D**) and *S. typhimurium*. Later, bacterial burden was estimated in the (**B**,**C**) spleen; (**D**) liver. Bar graph depicts the CFU/gm weight of the organs denoted as mean ± SD. (**E**) *Mtb* infected nDCs were cultured in the presence of iNOs inhibitor for 24 h. Intracellular survival was studied by CFU plating after 24 h. Results shown as mean ± SD are representative of 3 independent experiments. *p < 0.05, **p< 0.01.

Since nDCs expressed activated phenotype, we were curious to know whether they also displayed better ability to restrict the intracellular proliferation of pathogens. We observed significantly lesser survival of *Mtb* in nDCs, as supported by reduced number of CFUs compared to cDCs (Fig. 5A). It is important to mention here that the bar graphs depict reduction in the growth of intracellular bacterium at 24h; normalized with the difference in *Mtb* uptake by nDCs after 4h of infection. Thus, proving that the change observed is due to restriction in the growth of *Mtb* and not owing to differential uptake.

The functionality of nDCs was ultimately examined *in vivo* in the experimental model of TB. The nDCs were adoptively transferred into mice, followed by infection with *Mtb.* As compared to cDCs, these animals showed significant (p < 0.01) decline in the *Mtb* burden in the spleen (Fig. 5B). Activation of T cells in *Mtb* model takes 14–15d for their effector functions. Therefore, to show the role of nDCs in reduction of the bacterial burden, *Mtb* challenged mice were sacrificed after 10d of infection.

We were inquisitive to know whether the observed phenomenon was restricted to *Mtb* or to other pathogens, as well. Intriguingly, we noted significant decline in the CFUs of *Salmonella typhimurium* in the liver (p < 0.01) and spleen (p < 0.05) upon adoptive transfer of nDCs in mice (Fig. 5C,D); suggesting that the killing potency of nDCs was not restricted to *Mtb* alone. In addition, we further confirmed the involvement of NO in killing of *Mtb. Mtb* infected nDCs were cultured in the presence of iNOs inhibitor for 24 h. Interestingly, we observed the restoration of *Mtb* growth in the nDCs (Fig. 5E). Thus, ascertains the involvement of NO in restricting the growth of intracellular pathogens.

### Adoptive transfer of nDCs leads to notable release of IFN-γ and IL-17 by T cells

**Figure 6:**
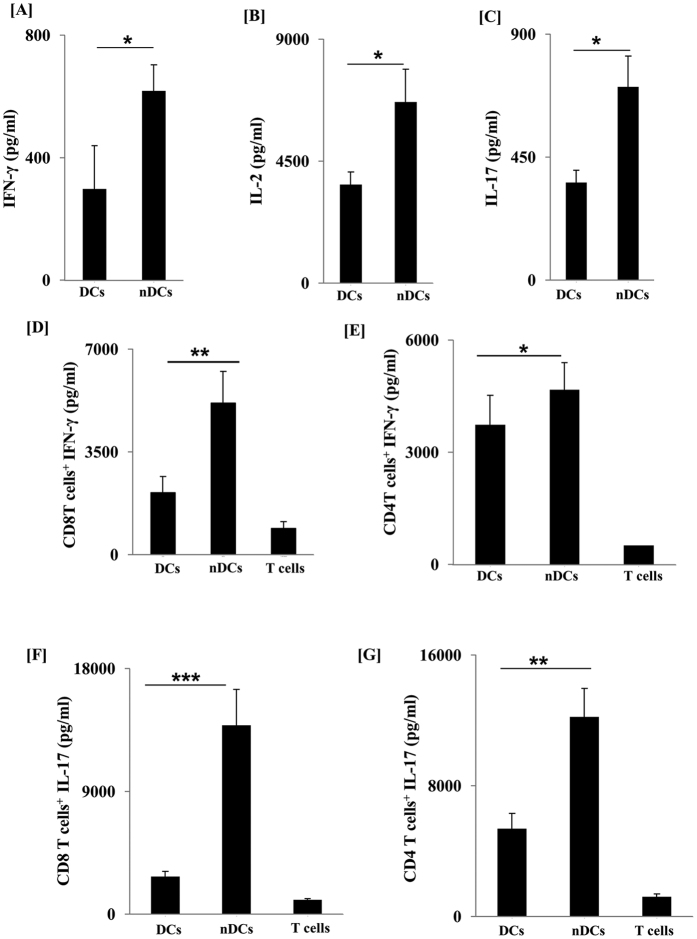
nDCs promoted release of IFN-γ and IL-17 by T cells. *In vivo* priming with nDCs: nDCs loaded with OVA were adoptively transferred into mice. After 7 d, animals were sacrificed and cells isolated from lymph nodes were cultured with OVA for 24 h. Later, the cytokines were quantified in the SNs by ELISA (**A**) IFN-γ; (**B**) IL-2; (**C**) IL-17. Data shown are representative of two independent experiments with 3 mice/group. *In vitro* priming with nDCs: (**D**–**G**) nDCs loaded with OVA were co-cultured with purified CD4 T cells and CD8 T cells isolated from the lymph nodes of OVA immunized mice. Later, secretion of IFN-γ and IL-17 by (**D**,**F**) CD8 T cells and (**E**,**G**) CD4 T cells in the SNs was quantified by ELISA. Data shown as mean ± SD are representative of 3 independent experiments. *p < 0.05, **p < 0.01, ***p < 0.001.

Activation of T cells by DCs is a foremost proof of their functionality^21^. To evaluate their T cell priming capability, we carried out the adoptive transfer of nDCs loaded with OVA to OVA-sensitized mice. Interestingly, we observed that the inoculation of OVA loaded nDCs markedly increased the production of IFN-γ (p < 0.05), IL-2 (p < 0.05) and IL-17 (p < 0.05) (Fig. 6A–C) than control DCs loaded with OVA. These results signify the optimum activation capacity of DCs.

### nDCs showed better potency to stimulate T cells

Next we assessed the ability of nDCs to *vis-a-vis* stimulate CD4 T cells and CD8 T cells. The T cells isolated from the spleen and lymph nodes of OVA sensitized mice were co-cultured with OVA pulsed-DCs. We observed that nDCs remarkably enhanced the activation of both CD4 T cells and CD8 T cells, as evidenced by substantially greater production of IFN-γ and IL-17 than cDCs (Fig. 6D–G). Thus, illustrates that nDCs acquire enhanced potential to activate T cells.

### nDCs maturation and activation is mediated via IFN-αβ signaling

**Figure 7:**
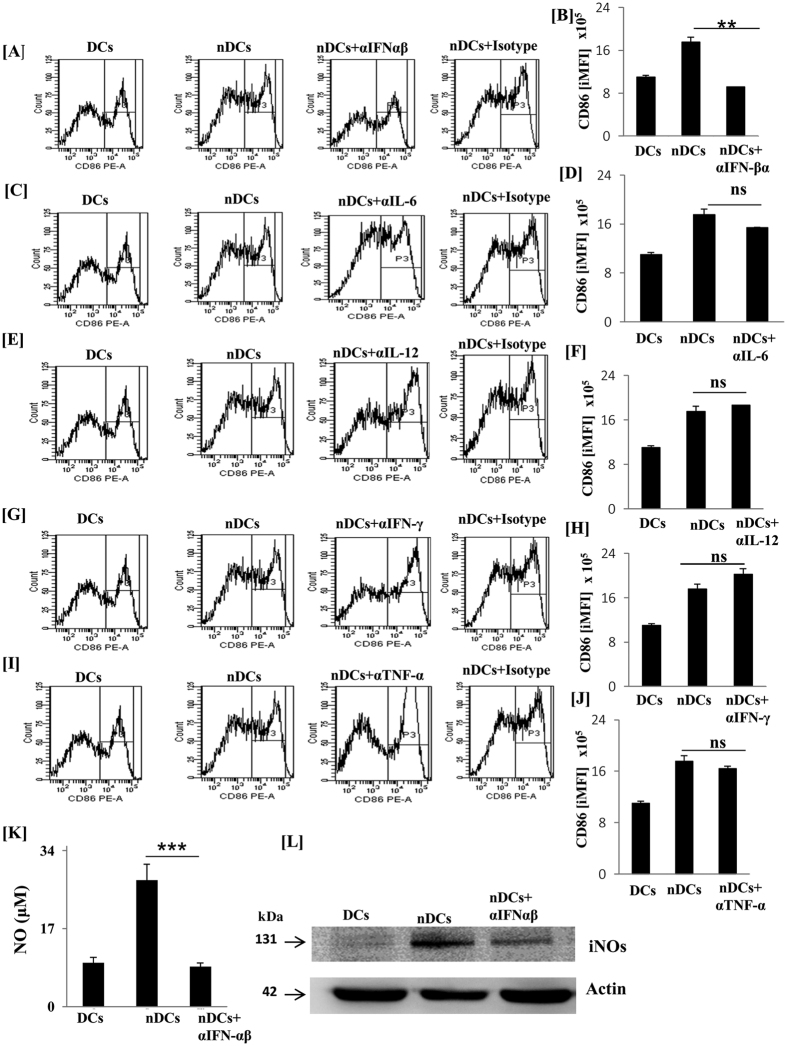
Signaling of NOD-2 induced the differentiation of BMCs to nDCs through IFN-αβ signaling. nDCs were differentiated in the presence of blocking/neutralizing Abs to (**A**,**B**) IFN-αβR; (**C**,**D**) IL-6; (**E**,**F**) IL-12; (**G**,**H**) IFN-γ; (**I**,**J**) TNF-α. (**A**) Later, nDCs were stained for the CD86 expression by flow cytometry. For analysis of CD86, cells were gated on SSC^+^ and CD11c^+^cells after the FSC and SSC gate was set. Bar graphs depict the integrated MFI (iMFI) for the CD86 expression. (**K**,**L**) nDCs cultured in the presence of blocking Abs to IFN-αβR showed decrease in the production of NO in the SNs and iNOs expression in whole cell lysate. Results depicted as mean ± SD are representative of 3 independent experiments. **p < 0.01, ***p < 0.001 ns: non-significant.

Ultimately, it was important for us to determine the NOD-2 driven mechanism responsible for the enhanced activity of nDCs. Cytokines like IL-6, IL-12, TNF-α and IFN-γ are known to regulate the differentiation and activation of DCs^10^,^22^. Noteworthy, NOD-2 recently has been shown to trigger the release of type-1 IFNs^23^. Further, type-1 IFNs have been shown to induce the maturation of DCs^24^. Taking into consideration of these facts, nDCs were differentiated in the presence of neutralizing/blocking Abs against IL-6, IL-12, IFN-γ, TNF-α and IFN-αβR. Remarkably, we observed that nDCs incubated with anti-IFN-αβR failed to acquire matured phenotype, as proven by significant (p < 0.01) downregulation in the expression of costimulatory molecule CD86 (Fig. 7A,B). In contrast, neutralization of IL-6, IL-12, IFN-γ and TNF-α showed no change (Fig. 7C–J). Likewise, blocking of IFN-αβR showed reduction (p > 0.001) in the NO production. This observation was corroborated by downregulation in the yield of iNOs by Western blotting (Fig. 7K,L).

### Triggering of DCs through NOD-2 stimulates Stat-1 and Stat-4 and inhibits SOCS signaling pathway

**Figure 8:**
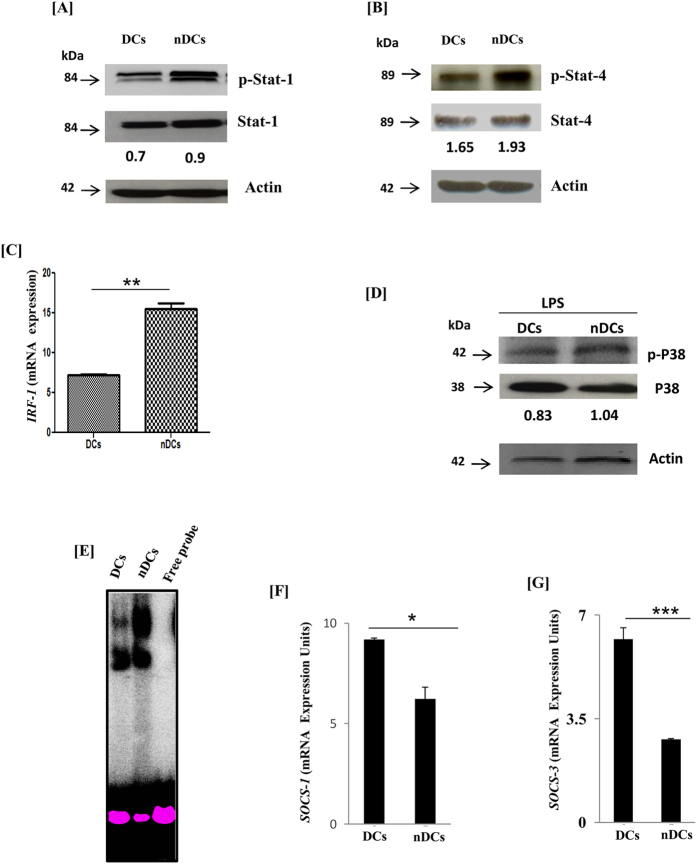
STAT pathways regulate the differentiation of nDCs. nDCs were harvested on 6 d and later protein extract was subjected to Western blotting using Abs against tyrosine phosphorylated (**A**) p-stat-1; (**B**); pstat-4. Membrane was than striped and re-probed with Abs to stat-1, stat-4 and β-actin. (**C**) mRNA expression was studied for IRF-1 by RT-qPCR; (**D**) nDCs stimulated with LPS were lysed and protein extracts were probed with anti-p-p38 Abs. Membrane was then striped and re-probed with anti-p38 and β-actin Abs. (**E**) Nuclear extract of nDCs was subjected to the nuclear translocation of NF-кβ by EMSA. (**F**,**G**) Expression of SOCS-1 and SOCS-3 was studied using nDCs by RT-qPCR. The data in the insets of the figures represent the fold change in phosphorylation (**A**,**B**,**D**). Data shown as mean ± SD are representative of two independent experiments. *p < 0.05, **p < 0.01, ***p < 0.001.

To gain further insight into the molecular mechanism underlying nDCs maturation and activation, we monitored the involvement of STAT signaling pathway, since it regulates the genes involved in the differentiation and maturation of DCs^25^,^26^. nDCs expressed higher phosphorylation of Stat-1 and Stat-4 than conventional DCs (Fig. 8A,B). Phosphorylation of Stat-1 induces the maturation of DCs^27^,^28^. Further, enhancement in the expression of Stat1 target gene IFN regulatory factor 1 (IRF1) was also noted (Fig. 8C). IRF-1 is known to induce the maturation and activation of DCs^29^. In addition, nDCs upon stimulation with LPS showed better phosphorylation of p38 molecules, which is also responsible for the activation of DCs (Fig. 8D)^30^. Furthermore, nDCs showed better nuclear translocation of NF-κβ (Fig. 8E). Suppressors of cytokine signaling (SOCS) molecules negatively regulate the expression of Stats to downregulate the activity of DCs^28^. Interestingly, nDCs showed reduction in the mRNA expression of SOCS-1 and SOCS-3 (Fig. 8F,G). These results collectively indicate that the higher expression of Stat-1 and Stat-4 along with downregulation of SOCS-1 and SOCS-3 is a mechanism responsible for improved function of nDCs.

## Discussion

Bone marrow cells consist of macrophage and DC precursors, which give rise to circulating monocytes. During inflammation, monocytes migrate to the site of infection and differentiate into DCs and macrophages. Effect of different cytokines in skewing monocytes to DCs or macrophages is well established. Since bone marrow precursors express copious amount of PRRs, it is very likely that the interaction of these receptors with pathogenic moieties will influence their differentiation. Stimulation through NOD-2 efficaciously differentiates human monocytes to DCs in experimental model of leprosy and substantially improves their ability to cross-present antigen to CD8 T cells^15^.

In the current study, we have demonstrated a novel role of NOD-2 signaling in promoting the development of highly immunogenic nDCs. nDCs showed following major features: i) enhanced activation and maturation phenotype; ii) augmented bactericidal activity was through increased iNOs expression; iii) efficiently presented antigen to CD4 T cells and CD8 T cells; iv) improved capacity to inhibit *in vivo* growth of intracellular pathogens; v) the mechanism responsible for immunogenicity of nDCs was dependent on IFN-αβ signaling pathways, leading to the activation of Stat-1 and Stat-4.

DCs functionality is determined by their immature and mature status^31^. Immature DCs are characterized by their ability of higher antigen uptake and lack the requisite signals for T cells activation. However, matured DCs lose their ability to uptake antigen. Further, they perform highly specialized function of priming T cells and therefore evoke adaptive immune response. nDCs exhibited augmented efficiency of antigen uptake and stimulation of T cells.

DCs differentiated in the presence of various TLR agonists are defective in their function^14^. Further, continuous exposure of DCs with antigen induces tolerization. Interestingly, unlike LPS (agonist of TLR-4), NGMDP (agonist of NOD-2) promoted the development of activated DCs^14^. Further, nDCs responded efficiently to different TLR agonists and upregulated the expression of costimulatory molecules and produced IL-6 and IL-12. Costimulatory molecules play a decisive role in deciding the anergy or activation of T cells^32^,^33^. IL-6 is a proinflammatory cytokine that is required at the time of infection and recruitment of cells to the inflammatory site. IL-12 is a hallmark cytokine to induce Th1 immunity^34^. DCs are inferior in bactericidal mechanism and restricting the growth of intracellular pathogens^2^. In contrast, nDCs chiefly activated Th1 cells and Th17 cells, exhibited enhanced bactericidal activity and were highly potent in killing *Mtb*. Further, they released more NO and displayed augmented levels of iNOs.

NOD-2 triggers IFN-αβ production, which is a classical activator of STATs and phosphatidyl-inositol-3’ kinase pathways^23^,^35^. Further, induction of interferon and STATs target genes are important components involved in DCs maturation and activation^27^. Noteworthy, nDCs showed maturation and activation involving phosphorylation of Stat-1, Stat-4 and p38 and induction in the expression of Stat-1 target gene IRF-1 and NF-κB. Furthermore, blocking of IFN-αβ resulted in the failure of nDCs to acquire highly activated phenotype, as evidenced by decrease in CD86 expression and NO release. STAT pathways are under the negative regulation of SOCS proteins. Importantly, NOD-2 triggered DCs showed decrease in mRNA expression of SOCS-1 and SOCS-3 proteins. These results suggest that NOD-2 signaling activates STATs mediated component of DCs maturation by inducing IFN-αβ signaling.

DCs are professional APCs with a capability to prime naive T cells and initiate specific immune responses. Their role is also deciphered in vaccination strategies, especially for anti-tumor immunotherapies^36^. Consequently, antigen-loaded autologous DCs have reached the stage of human clinical trials. These studies have been made possible by obtaining large number of DCs through *ex vivo* generation of monocytes-driven DCs. Thus, it is imperative to identify factors that enhance the yield of DCs. It was extremely exciting for us to note that signaling through NOD-2 substantially expanded the pool of DCs with improved efficiency to stimulate innate immunity, as well as adaptive immunity by activating Th1 cells, Th17 cells and CD8 T cells. These cells play cardinal role in protecting against *Mtb*. Innate immunity is crucial in combating early infections, whereas adaptive immunity is necessary at later stage.

In essence, our study for the first time demonstrates a novel role of NOD-2 signaling in differentiating bone marrow precursors to highly immunogenic DCs. These DCs efficiently activated both innate as well as adaptive immunity that controlled the growth of *Mtb*. Finally, this strategy of stimulating DCs through NOD-2 may be extremely useful in bolstering immunity for treating infectious diseases.

## Material and Methods

### Animals

Female C57BL/6 mice, 6–8 weeks (20 ± 2 gm) were procured from the animal house, Institute of Microbial Technology, Chandigarh, India.

### Ethics statement

All experiments were approved by the Institutional Animal Ethics Committee of Institute of Microbial Technology and performed according to the National Regulatory Guideline issued by Committee for the Purpose of Supervision of Experiments on Animals (No. 55/1999/CPCSEA), Ministry of Environment and forest, Govt. of India.

### Antibodies and reagents

All the standard chemicals and reagents were purchased from Sigma (St. Louis, MO) or unless mentioned. Abs and recombinant cytokines were purchased from BD Biosciences (San Diego, CA). TLR-4 ligand (LPS EK ultrapure), NOD-2 ligand (N-glycolyl MDP), TLR-9 ligand (CPG-ODN), TLR-7 ligand (imiquimod), TLR-3 ligand (Poly I:C) were procured from Invivogen (San Diego, CA). Abs to iNOs was purchased from Abcam (Cambridge, UK).

### Culture of BMCs derived DCs and their stimulation through NOD-2 and TLR-4

BMCs were cultured according to Lutz *et al*.^37^. Briefly, BMCs were flushed aseptically from the femurs and tibia. The cells were cultured in RPMI-1640 + FCS-10% (GIBCO, Grand Island, NY) supplemented with penicillin (100 U/ml), streptomycin (100 mg/ml), and L-glutamine (100 mM), and granulocyte-macrophage colony-stimulating factor (GMCSF) (2 ng/ml) and rIL-4 (4 ng/ml) in the presence of different concentrations of NGMDP (3-12 μg/ml) for 6 d. Cultures were maintained in a humidified atmosphere, CO_2_ (5%) at 37 °C. The medium containing GMCSF + IL-4 + NGMDP was replenished on 3 d.

For neutralization experiments, neutralizing antibody for IL-6 (20 μg/ml), IL-12 (20 μg/ml), TNF-α (10 μg/ml), IFN-γ (10 μg/ml) and blocking antibody for IFN-αβR (20 μg/ml) was added in culture along with GMCSF + IL-4 + NGMDP on the 1 d, and replenished on 3 d.

### *In vitro* infection with mycobacteria

*Mtb* (H37Ra or H37Rv) were grown to mid log phase in 7H9 broth supplemented with glycerol, tween-80, albumin-10%, dextrose and catalase enriched medium. Liquid culture was grown for 12 d and aliquots were stored at −80 °C till use. For infection, aliquots were rapidly thawed, washed twice with PBS (1X) and re-suspended in RPMI-FCS-10%. Bacteria were passed through a syringe and added to nDC cultures at a multiplicity of 1:5 infection and incubated for 4 h. Later, cells were washed extensively to remove extracellular bacteria.

### CFU determination

The bacterial growth was quantified 24 h after infection. The cell supernatants (SNs) were removed and saponin (0.1%) was added to lyse the cells and plating was done with 10 fold serial dilution on 7H11 agar plates and later kept at 37 °C in a humidified atmosphere. The colonies were enumerated after 3 wk.

### Immunofluorescence staining

nDCs were harvested at indicated time and re-suspended in the flowcytometry buffer (FCS-2%, sodium azide-2 mM in PBS). To inhibit non-specific staining, cells were incubated with anti-CD16/32 for 25 min/4 °C, washed and stained with fluorochrome conjugated Abs specific for phenotypic markers CD80, CD86 and CD40 and their isotype-matched control Abs. The cells were washed, fixed with paraformaldehyde-1X. Data were collected using FACS Aria and analyzed with BD DIVA software.

### Nitric oxide (NO) production

NO was estimated in the culture SNs by Griess method at time mentioned in legends. Briefly, SNs (50 μl) were incubated with an equal volume of Griess reagent for 5 min/RT. Later, absorbance was measured at 550 nm. For NO inhibition assay, *Mtb* infected DCs were cultured in the presence or absence of NO inhibitor (N-monomethyl-L- arginine) for 24 h.

### Cytokines estimation by ELISA

Cytokines *viz.* IL-6, IL-12 and IFN-γ were detected in the culture SNs at the indicated time point by ELISA, according to manufacturer’s instruction (BD Biosciences, San Diego, CA).

### Antigen uptake

nDCs were harvested, washed, and then pulsed with HRP (50 μg/ml). Pulse-chased experiments were conducted for 30–60 min and antigen uptake was arrested by adding chilled PBS (1X) followed by transferring cells on ice. The cells were washed extensively with ice cold PBS-FBS-1%. The cells were lysed using Tris-HCl (10 mM) and Triton X-100 (0.05%) for 30 min on ice, with intermittent vortexing. Intracellular HRP was estimated colorimetrically in the cell lysates using OPD-H_2_O_2_ chromogen-substrate. The cells maintained at 4 °C were used as control. HRP activity in test samples was suitably normalized with controls.

For confocal analysis, nDCs were infected with GFP-*Mtb* (H37Ra) at (MOI 1:5) for 4 h. The cells were washed extensively (4X) with ice cold PBS (1X) and fixed with paraformaldehyde (4%). The cells were washed and placed on poly-L-lysine coated cover slips and imaged using Zeiss Confocal Laser Microscope. Results were examined by image analysis software.

For the estimation of bacterial uptake, nDCs were harvested and *Mtb* infection was given at MOI (1:5) for 4 h. It was followed by lysis of cells with 0.1% saponin and plating was done with 10 fold serial dilution on 7H11 agar plate. The colonies were enumerated by CFUs, 3 wk after incubation at 37 °C/5% CO_2_.

### Real Time PCR

To quantify RNA levels of nDCs, total RNA was isolated by Trizol reagent according to the manufacturer’s instruction (Invitrogen, Carisbad, CA). RNA was quantified with the help of NanoDrop spectrophotometer. A260/A280 ratios of all samples were in the range of 1.90 to 2.00. Intactness of RNA samples was determined with the help of formaldehyde denaturing agarose gel electrophoresis. DNA contamination from RNA samples was removed by using amplification grade DNase. Briefly, RNA samples (1 μg) were incubated with DNase (1 U) for 15 min in the reaction buffer. After incubation, DNase activity was terminated by adding stop solution. Further, the samples were heated to 70 °C/10 min to inactivate DNase activity. Results were represented as relative expression (fold). Analysis was done by comparative Ct method. Real time PCR and data analysis were done by Realplex Mastercycler (Eppendorf, Hamburg, Germany).

Following primers were used for the quantification:

TGF-β Fwd 51-TGACGTCACTGGAGTTGTACGG-31.

Rev 51-GGTTCATGTCATGGATGGTGC-31.

β-actin: Fwd 51-AGAGGGAAATCGTGCGTGAC-31.

Rev 51-CAATAGTGATGACCTGGCCGT-31.

IRF1 Fwd 51-ATAACTCCAGCACTGTCACCGTG-31.

Rev 51-ATCCTCGTCTGTTGCGGCTT-31.

IFN- β Fwd 51-GCACTGGGTGGAATGAGACT-31.

Rev 51-AGTGGAGAGCAGTTGAGGACA-31.

### Western blotting

For iNOs, nDCs harvested on 6 d of culture were stimulated with LPS for 24 h or infected with *Mtb* for 4 h. *Mtb* infected DCs were washed to remove extracellular bacteria and cultured for 24 h. After 24 h, cells were washed, and lysed in lysis buffer (RIPA buffer, protease and phosphatase inhibitor cocktail). In SNs, proteins were estimated and equal concentration was subjected to SDS-PAGE. After transfer to nitrocellulose membrane and subsequent blocking, the membranes were immunoblotted with Abs against iNOs and actin (loading control). Blots were developed using chemiluminescence kit (Amersham Pharmacia Biotech, Buckinghamshire, UK). Blots were scanned using phosphoimager (Fujifilm, Tokyo, Japan) and images analyzed employing MultiGuage software. pStat-1, pStat-4 and p38 were detected in the lysate of nDCs harvested on 6 d of culture following the same protocol as mentioned above. The fold change in the phosphorylation of Stat-1, Stat-4 and p38 was calculated as a ration between phosphorylated and un-phosphorylated forms.

### Adoptive transfer of nDCs to study bactericidal activity

nDCs were adoptively transferred into mice. After 5 d, mice were challenged with *Mtb (i.v*) and *Salmonella typhimurium (S. typhimurium*) (i.p) and sacrificed on 10 d and 3 d, respectively. Later, *Mtb* burden was estimated in spleen and *S. typhimurium* in the liver and spleen.

### *In vivo* polarization of T cells by nDCs

The polarization of T cells was done as mentioned elsewhere^38^. Mice were injected s.c. with OVA (100 μg) in aluminum hydroxide (2 mg) dissolved in PBS (0.2 ml). Ten days later, nDCs incubated for 2 h with OVA (100 μg/ml) were adoptively transferred s.c. into OVA-primed mice. On 7 d, the mice were sacrificed and lymph nodes were isolated and single cell suspension was prepared. These cells (2 × 10^5^/well) were then cultured with OVA (100 μg/ml) for 24 h. Later, polarization of T cells was monitored by quantification of IFN-γ, IL-17 and IL-2 in the SNs by ELISA.

### *In vitro* activation of CD4 T cells and CD8 T cells by nDCs

Mice were sensitized by s.c. injection of OVA (100 μg). After 7 d, lymph nodes were isolated and single cell suspension was prepared to purify CD4 T cells or CD8 T cells using magnetic associated cells sorting (MACS). Purified CD4 T cells or CD8 T cells were co-cultured with OVA loaded DCs at a ratio of 10:1 (T cells: DCs) for 5 d^38^. Later, IFN-γ and IL-17 were estimated in SNs by ELISA.

### Statistics

Level of significance was performed by statistical analysis using unpaired student’t test’ by graph pad prism software.

## Additional Information

**How to cite this article**: Khan, N. *et al*. Triggering through NOD-2 Differentiates Bone Marrow Precursors to Dendritic Cells with Potent Bactericidal activity. *Sci. Rep.* **6**, 27263; doi: 10.1038/srep27263 (2016).
